# Hippocampal gene expression patterns in Sevoflurane anesthesia associated neurocognitive disorders: A bioinformatic analysis

**DOI:** 10.3389/fneur.2022.1084874

**Published:** 2022-12-06

**Authors:** Weiwei Li, Qijun Yi, Huijian Shi

**Affiliations:** ^1^Department of Anesthesiology, The Second Affiliated Hospital of the Shandong First Medical University, Taian, China; ^2^Department of Oncology, The Second Affiliated Hospital of the Shandong First Medical University, Taian, China

**Keywords:** Sevoflurane, anesthesia, neurocognitive disorders, molecular mechanisms, bioinformatics

## Abstract

**Background:**

Several studies indicate general anesthetics can produce lasting effects on cognitive function. The commonly utilized anesthetic agent Sevoflurane has been implicated in neurodegenerative processes. The present study aimed to identify molecular underpinnings of Sevoflurane anesthesia linked neurocognitive changes by leveraging publically available datasets for bioinformatics analysis.

**Methods:**

A Sevoflurane anesthesia related gene expression dataset was obtained. Sevoflurane related genes were obtained from the CTD database. Neurocognitive disorders (NCD) related genes were downloaded from DisGeNET and CTD. Intersecting differentially expressed genes between Sevoflurane and NCD were identified as cross-talk genes. A protein-protein interaction (PPI) network was constructed. Hub genes were selected using LASSO regression. Single sample gene set enrichment analysis; functional network analysis, pathway correlations, composite network analysis and drug sensitivity analysis were performed.

**Results:**

Fourteen intersecting cross-talk genes potentially were identified. These were mainly involved in biological processes including peptidyl-serine phosphorylation, cellular response to starvation, and response to gamma radiation, regulation of p53 signaling pathway, AGE-RAGE signaling pathway and FoxO signaling. Egr1 showed a central role in the PPI network. Cdkn1a, Egr1, Gadd45a, Slc2a1, and Slc3a2 were identified as important or hub cross-talk genes. Among the interacting pathways, Interleukin-10 signaling and NF-kappa B signaling enriched among Sevoflurane-related DEGs were highly correlated with HIF-1 signaling enriched in NCD-related genes. Composite network analysis showed Egr1 interacted with AGE-RAGE signaling and Apelin signaling pathways, Cdkn1a, and Gadd45a. Cdkn1a was implicated in in FoxO signaling, PI3K-Akt signaling, ErbB signaling, and Oxytocin signaling pathways, and Gadd45a. Gadd45a was involved in NF-kappa B signaling and FoxO signaling pathways. Drug sensitivity analysis showed Egr1 was highly sensitive to GENIPIN.

**Conclusion:**

A suite of bioinformatics analysis revealed several key candidate hippocampal genes and associated functional signaling pathways that could underlie Sevoflurane associated neurodegenerative processes.

## Background

Sevoflurane is currently one of the most commonly applied anesthetic agents with a high safety record of over two decades (1, 2). It is a volatile anesthetic, which have a low tissue and blood-gas solubility and partition coefficient as compared to traditional inhaled anesthetics (3). These support a fast uptake, control of depth, and fast elimination resulting in faster recovery time and shorter periods of respiratory depression (4, 5). Furthermore, it has also shown cardio protective effects when used in cardiac surgery and also in other organs (6, 7). Therefore it is frequently applied in both induction and maintenance of general anesthesia.

Cognitive deficits after general anesthesia and surgery have been observed since a long time (8, 9). Various forms of short and long-term cognitive disturbances after anesthesia and surgery have been extensively documented. These include postoperative delirium and postoperative cognitive dysfunction (9, 10). Postoperative cognitive dysfunction can lead to long-term impairments multiple domains including executive function, visual-spatial and verbal memory, processing speed (11).

Increasingly, research has shown that general anesthetics can induce long lasting brain changes marked by altered tissue morphology and function, with the elderly and pediatric groups being the most vulnerable. Experimental studies have revealed several mechanistic aspects, including apoptotic cell death, impairment of neurogenesis and synaptic loss (12). Multiple molecular mechanisms have been implicated in general anesthetic mediated neurotoxicity, among which the role of pro-BDNF/p75/RHOA axis leading to actin depolymerization, synapse loss and apoptosis has been highlighted (13). Additionally, the role of increased mitochondrial complex leading to reactive oxygen species production and caspase activation has been identified as a mechanism leading to neuronal apoptosis (14).

Repeated Sevoflurane anesthesia has been shown to induce increased neuroinflammation marked by rise IL-6 levels and aberrant AKT signaling (15). Sevoflurane anesthesia was also found to induce phosphorylation of the tau protein, causing activation of GSK3β signaling and cognitive damage (16). Other purported mechanisms include increased α5GABAAR activity (17). Sevoflurane was found to induce higher neurotoxocity, caspase-mediated apoptosis and amyloid accumulation in Alzheimer's disease (AD) transgenic mice, suggesting higher susceptibility (18). Particularly, aberrant functioning of the hippocampus has been implicated in anesthesia induced cognitive dysfunction (19).

Despite available experimental data, many critical gaps remain in the understanding of general anesthetic induced neurocognitive disorders (NCD). Among these, specific differences between different anesthetic agents remain unclear. Molecular mechanisms and functional signaling pathway aberrations that can underlie Sevoflurane anesthesia linked effects on NCD have not been comprehensively and systematically investigated. Secondary integrative utilization of available gene expression and other bioinformatic data can allow exploratory analyses to reveal novel candidate mechanistic pathways which can direct translational research. Therefore, in the present bioinformatic study, we hypothesized that mechanisms implicated in Sevoflurane anesthesia associated cognitive impairment could be explored by leveraging gene expression data related to Sevoflurane anesthesia and NCD to identify shared features.

## Materials and methods

### Sevoflurane anesthesia related dataset

For the Sevoflurane anesthesia related dataset, we downloaded GSE139220 (PRJNA578770) (20) from NCBI GEO (https://www.ncbi.nlm.nih.gov/geo/), which described Sevoflurane anesthesia related gene expression in the hippocampus of aged rats. The dataset consists of 3 Cases and 3 Controls, and the species was Rattus norvegicus. In addition, we retrieved 314 differentially expressed genes related to Sevoflurane anesthesia (Mus musculus) through literature (21) (PRJNA556843). The screening criteria was FDR < 0.05, |logFC| > 0) which identified 49 up-regulated and 265 down-regulated genes. We also obtained a Sevoflurane anesthesia-related gene expression dataset and Sevoflurane anesthesia-related pathway dataset from CTD (http://ctdbase.org/). Since human genes were obtained from the CTD database whereas mouse data was mainly used in this study, the “biomaRt” package in R project was used to convert gene names between species.

### Neurocognitive disorders (NCD) related datasets

We downloaded genes associated with neurocognitive disorders (NCD) from the DisGeNET (https://www.disgenet.org/) database (selected diseases named Mild Neurocognitive Disorders and neurocognitive disorders). In addition, we also obtained NCD-related genes and pathways from CTD. Next, we combined the NCD-related genes obtained from the two databases for subsequent analysis. Since the human genes were obtained from the two databases, and mouse data was mainly used in this study, the “biomaRt” package in R project was used to convert gene names between species.

### Preprocessing and differential expression analysis of the Sevoflurane anesthesia dataset

For GSE139220, we converted the probe ids in the chip to gene symbol based on the platform information. In performing the transformation, we screened the NCBI refseq database for annotated genes. Then we filtered the dataset by genes applying filtering rules: (1) If the expression value of the gene in more than 50% of the samples was 0, then we removed the gene. (2) If the same gene had multiple expression values in a certain sample, we deduplicated based on the average.

Differential expression analysis compares the expression values of different groups of samples in the dataset, and predicts whether genes are differently expressed between different groups. For GSE139220, we used the “limma” package in R project for the differential expression analysis. The parameters used were Case vs. Control. Genes with *P* value < 0.05 and |logFC| > 0 as were selected as differentially expressed genes (DEGs). A volcano plot depicted the distribution of differentially expressed genes.

### Cross-talk genes between Sevoflurane anesthesia and NCD

We merged the two sets of differentially expressed genes (DEGs) associated with Sevoflurane, and then intersected the merged DEGs with the Sevoflurane anesthesia-related genes obtained from CTD. The resultant overlapping genes were considered as the definitive Sevoflurane anesthesia-related DEGs. Next, we obtained the intersection of Sevoflurane anesthesia-related DEGs and NCD-related genes, to identify common DEGs shared by Sevoflurane anesthesia and NCD, namely, the cross talk genes.

Functional enrichment analysis was conducted using the ‘clusterprofiler' package in R to analyze enriched GO Biological process and KEGG pathways. At the same time, the human homologous genes corresponding to these cross-talk genes were extracted and functional enrichment analysis using GO Biological process and KEGG pathway was performed.

### Cross talk gene protein-protein interaction (PPI) network

To obtain the role of cross talk genes in protein networks, we extracted the interacting proteins of the cross talk genes from the STRING database (https://cn.string-db.org/). Among the proteins interacting with the cross talk genes, we further screened those that appeared in any two groups from the three datasets including Sevoflurane anesthesia related genes and the NCD genes. Next, we used Cytoscape (version 3.9.1) to construct a PPI network and explored the the topological properties of the network.

### Screening of hub cross-talk genes

We used LASSO (Least absolute shrinkage and selection operator) Logistic Regression to perform feature selection among the Cross talk genes. We first extracted the expression values of the cross-talk genes from GSE139220, and then based on the sample type, we used LASSO to build a model for feature screening. As these feature selected genes obtained by LASSO analysis could be considered to play important connecting roles between Sevoflurane anesthesia and NCD, they were considered as hub cross talk genes.

The expression values of the hub cross talk gene in GSE139220 were obtained and *t*-test was applied to verify significant group differences in the hub cross talk gene expression values. In addition, Pearson correlation coefficient was computed to analyze the correlation between the hub cross talk gene and other cross talk genes.

### Single sample gene set enrichment analysis (ssGSEA)

ssGSEA is a tool that calculates enrichment scores for pairing of each samples with a gene set and generates gene enrichment score for each sample. We obtained Sevoflurane anesthesia and NCD-related pathways from the CTD database from KEGG (https://www.kegg.jp/) and Reactome (https://reactome.org/). We download all pathways for Rattus norvegicus and the genes under the pathways from the KEGG and Reactome. Based on the pathway-gene dataset, we obtained all genes under the pathways related to Sevoflurane anesthesia and NCD, each. Then we used the GSVA package in R project to perform ssGSEA analysis on GSE139220, and calculated the abundance scores of Sevoflurane anesthesia and NCD-related pathways, each. For the pathways related to Sevoflurane anesthesia, we used the limma package in R to perform differential expression analysis based on the ssGSEA scoring results, and considered the pathways with *P* value < 0.05 as DE-pathways. For the NCD-related pathways, we performed correlation analysis between the NCD pathways and the DE-pathways of Sevoflurane anesthesia, and then screened the NCD pathway highly related to the DE-pathways (marked as NCD hub pathway).

### Hub cross talk gene and pathway correlation analysis

To further analyze the possible relationship and influence of Sevoflurane anesthesia on NCD in relation to gene transcriptome and function, we used Pearson correlation coefficient to analyze the relationship among hub cross talk gene, Sevoflurane anesthesia DE-pathways and NCD hub pathways.

### Hub cross talk gene and pathway complex functional network

We extracted genes (marked as Target genes) that interacted with the hub cross talk genes in the PPI network of the cross talk genes. Then, we extracted the Pathways related to hub cross talk genes and Target genes from the Pathways of Sevoflurane anesthesia and NCD. We integrated the relationship pairs composed of hub cross talk genes, Target genes and Pathways, and finally constructed a composite functional network with pathways and hub cross talk genes.

### Candidate drug prediction

We extracted the human homologous genes of the hub cross talk genes, and then downloaded drug-related genes (version 2022-Feb) from DGIdb (https://dgidb.genome.wustl.edu/). We screened the drugs targeting the hub cross talk genes and Target genes to identify the potentially useful candidate drugs for Sevoflurane associated cognitive impairment.

## Results

### Differential expression analysis

**Figure 1:**
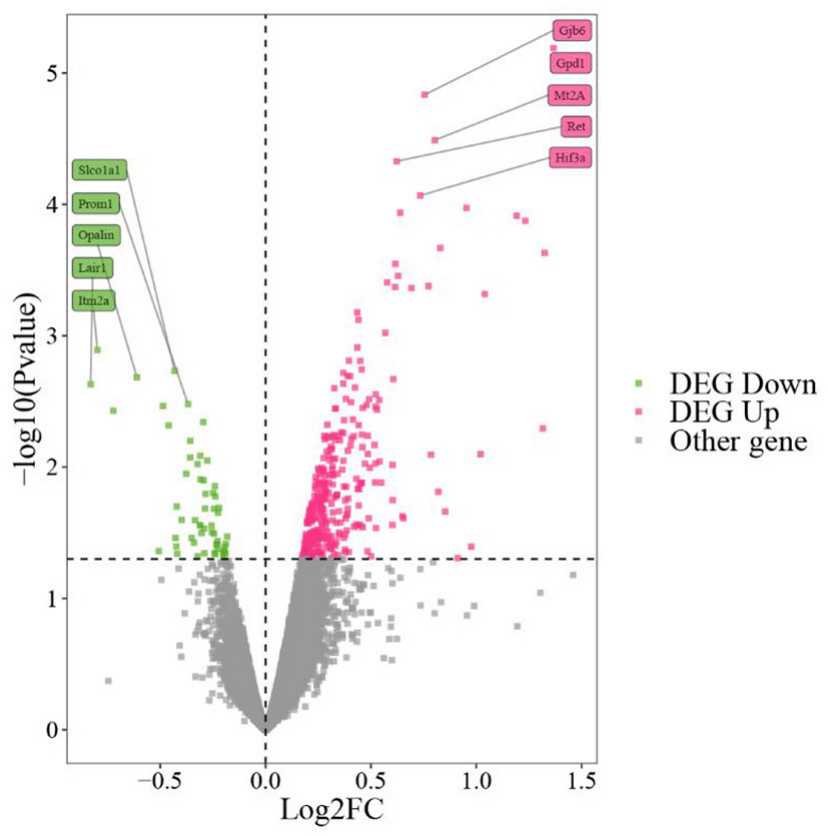
GSE139220 volcano map. The top 10 genes with the most significant *P* values are marked on the graph.

We performed differential expression analysis on GSE139220. We selected genes with *P* value < 0.05 and |logFC| > 0 as DEGs, where log2FC > 0 indicated an up-regulated gene, and log2FC < 0 indicated a down-regulated gene. Thus, we obtained 392 differentially expressed genes, including 335 up-regulated genes and 57 down-regulated genes (Figure 1).

### Cross-talk genes of Sevoflurane anesthesia and NCD

**Figure 2:**
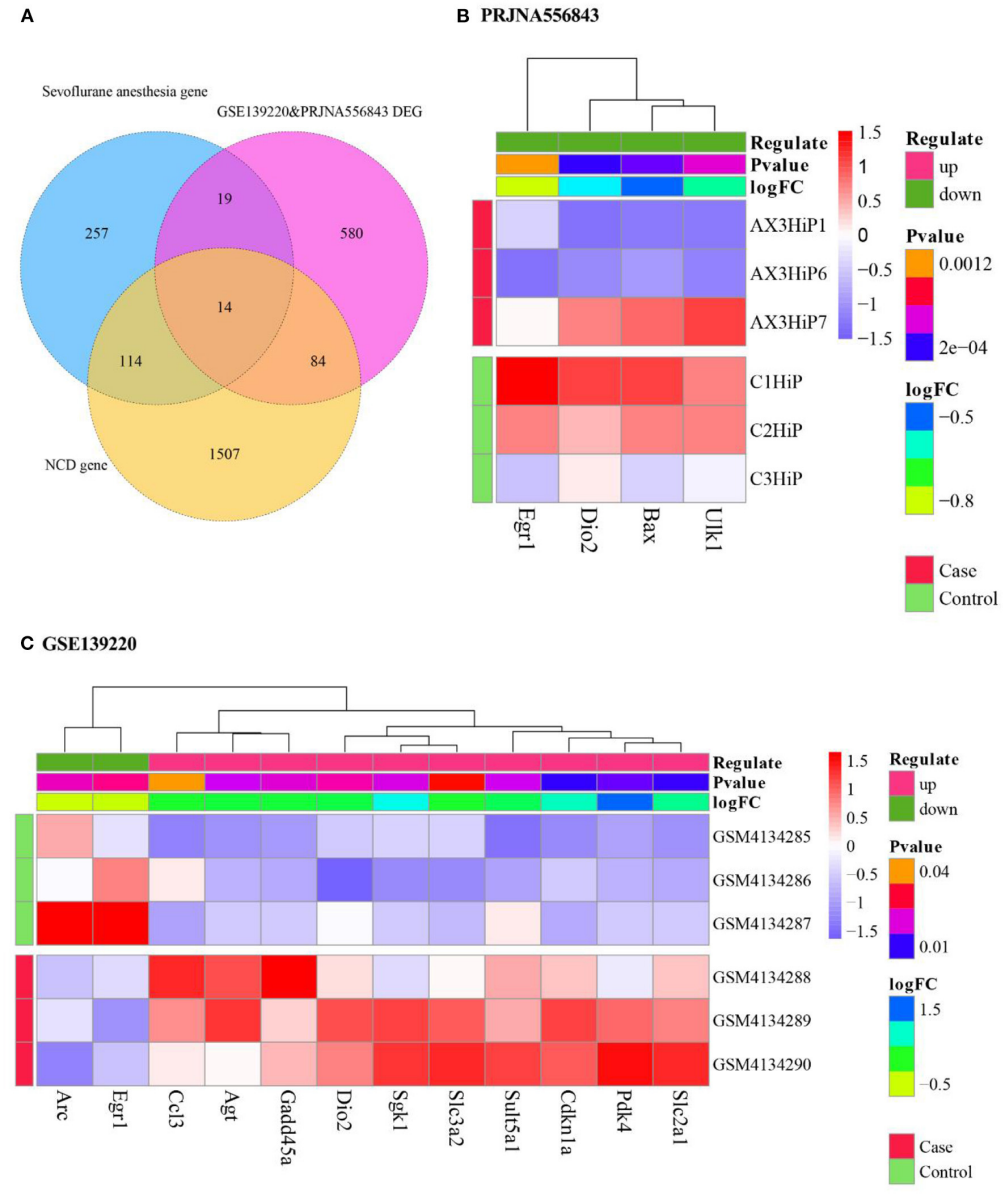
Cross talk genes linking Sevoflurane anesthesia and NCD. **(A)** Screening of cross-talk genes. PRJNA556843 differential gene; **(B)** heat map of cross-talk gene expression in PRJNA556843; **(C)** heat map of cross-talk gene expression in GSE139220.

We merged the 392 DEGs from GSE139220 (20) and the 314 DEGs from PRJNA556843 (21) and obtained 697 DEGs after deleting the duplicated genes. Next, we intersected the 697 DEG and Sevoflurane anesthesia-related genes from CTD databases to obtain 33 overlapping genes. Subsequently, from the intersection of 33 overlapping genes related to Sevoflurane anesthesia and NCD-related genes, we obtained 14 cross-talk genes (Figure 2A). The differential expression values of these of 14 cross-talk genes are shown in Table 1.

**Table 1:** Difference analysis results of 14 cross talk genes.

**Gene**	**Data**	**LogFC**	***P* value**	**Regulate**
Sult5a1	GSE139220	0.38249	0.0140901	Up
Slc3a2	GSE139220	0.2093867	0.0364218	Up
Slc2a1	GSE139220	0.5686387	0.0009515	Up
Sgk1	GSE139220	0.8205483	0.015407	Up
Pdk4	GSE139220	1.316298	0.0050749	Up
Gadd45a	GSE139220	0.2703933	0.0171574	Up
Cdkn1a	GSE139220	0.692644	0.0004328	Up
Ccl3	GSE139220	0.221027	0.0476096	Up
Arc	GSE139220	−0.421316	0.0199493	Down
Agt	GSE139220	0.2838157	0.0133006	Up
Egr1	GSE139220	−0.398263	0.0251983	Down
	PRJNA556843	−0.63057	0.0012029	Down
Dio2	GSE139220	0.3196857	0.0221334	Up
	PRJNA556843	−0.436048	6.91E-05	Down
Ulk1	PRJNA556843	−0.4822	0.000481	Down
Bax	PRJNA556843	−0.376606	0.0001761	Down

Then we extracted the expression values of 14 cross-talk genes in GSE139220 and PRJNA556843. 12 genes were expressed in GSE139220, and 4 genes were expressed in PRJNA556843. We used R's pheatmap package to draw a heatmap of gene expression in GSE139220 and PRJNA556843 (Figures 2B,C).

### Functional enrichment of cross talk gene

**Figure 3:**
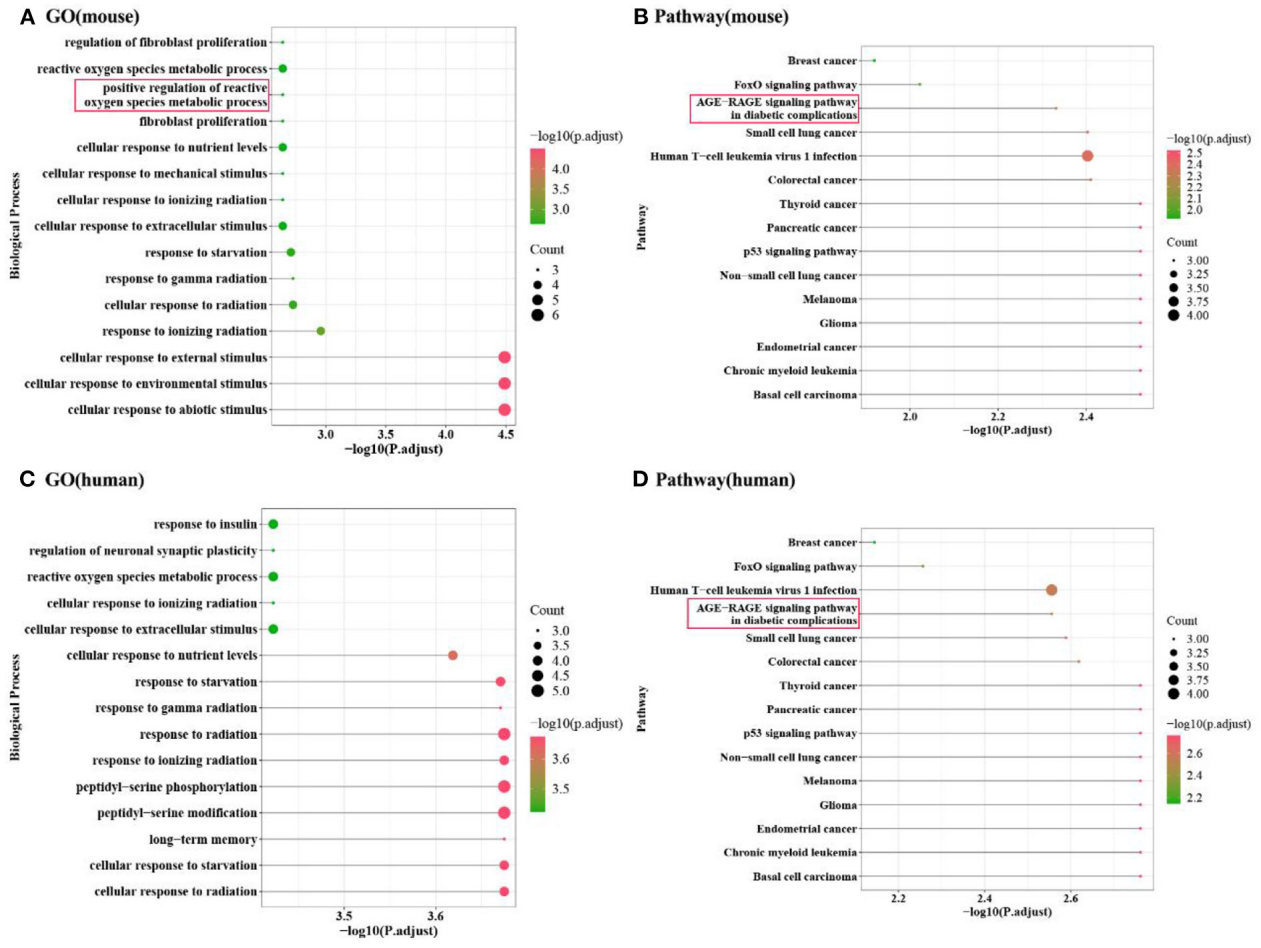
Enrichment analysis results of Cross talk gene. **(A,B)** Mouse cross-talk gene enrichment analysis in GO biological process and KEGG pathway; **(C,D)** Human homologous cross-talk gene enrichment analysis in GO biological process and KEGG pathway.

We used the clusterProfiler package in R to analyze the GO Biological process and KEGG pathway of mice for these 14 cross talk genes. We selected functions with *P*. adjust < 0.05 as significant and displayed the Top 20 functions (Figures 3A,B). In addition, we also performed GO Biological process and KEGG pathway analysis on the human homologous genes corresponding to the 14 cross talk genes, and selected *P*. adjust < 0.05 as a significant function (Figures 3C,D).

The results showed that the cross-talk genes were mainly involved in biological processes including peptidyl-serine phosphorylation, cellular response to starvation, and response to gamma radiation. In addition, cross-talk genes were involved in the regulation of p53 signaling pathway, AGE-RAGE signaling pathway in diabetic complications and FoxO signaling pathway.

### Cross talk gene PPI network

**Figure 4:**
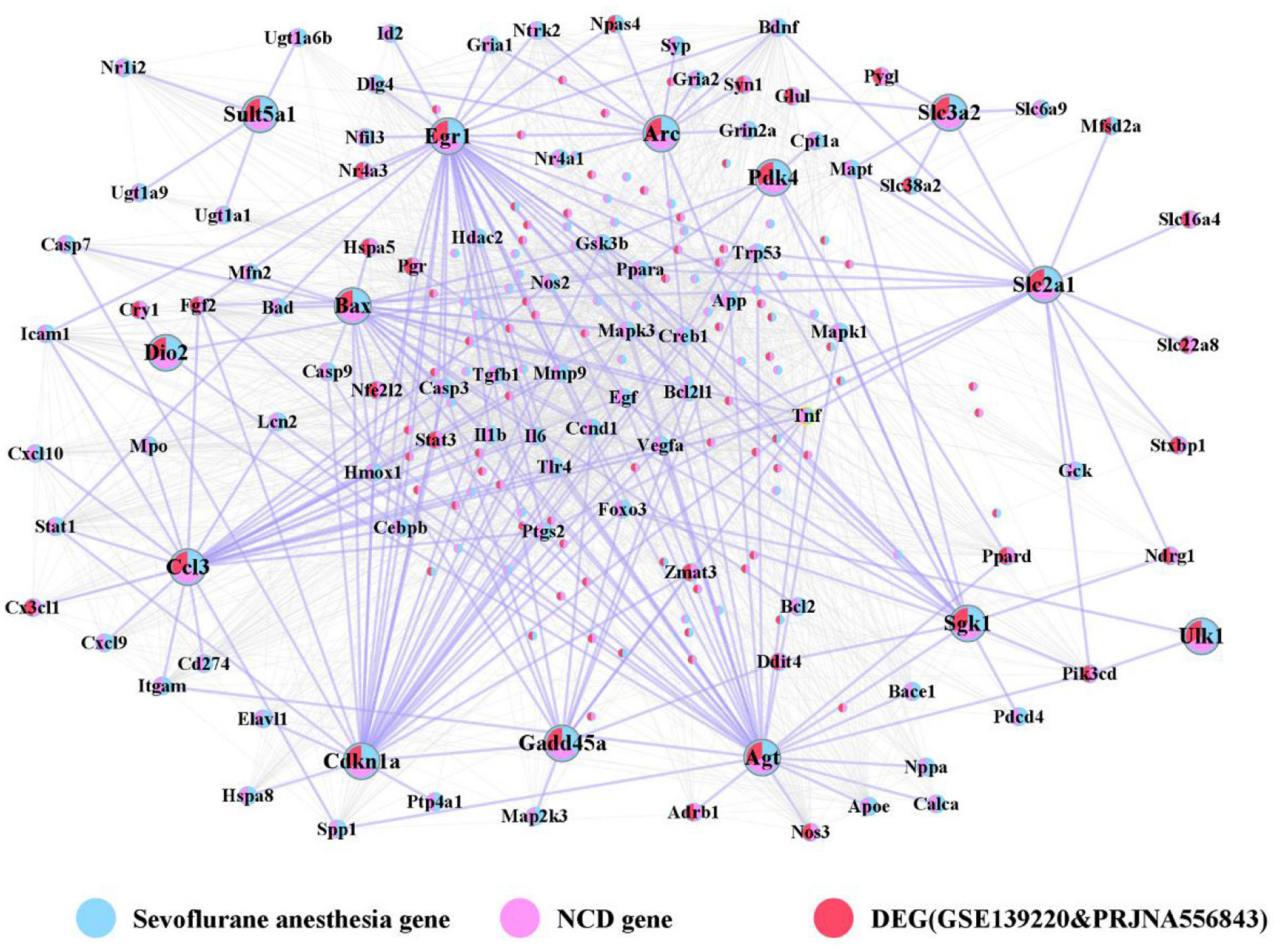
Cross talk gene PPI network. The network contains 210 nodes and 1,842 edges.

We obtained a total of 231 genes at the intersection of any two datasets from Figure 2A. Based on the String database, we extracted the cross-talk genes and the PPIs between these 231 genes, and constructed a PPI network for these relationship pairs (Figure 4).

Next, we analyzed the topological properties of the network, and extracted the topological properties of 14 cross talk genes. The results indicated a dominant role of Egr1 in the network (Table 2).

**Table 2:** The topology character of cross talk gene in PPI network.

**Gene**	**Degree**	**Average shortest** **pathlength**	**Betweenness** **centrality**	**Closeness** **centrality**	**Topological coefficients**
Egr1	40	2	0.009451	0.5	0.25731
Agt	34	2.052632	0.002564	0.487179	0.281965
Cdkn1a	33	2.105263	0.011983	0.475	0.287495
Ccl3	26	2.287081	3.08E-04	0.437238	0.354962
Bax	22	2.220096	6.94E-04	0.450431	0.344156
Slc2a1	17	2.215311	0.034202	0.451404	0.208267
Sgk1	16	2.311005	0.00142	0.432712	0.283062
Arc	13	2.430622	5.05E-04	0.411417	0.293956
Gadd45a	13	2.430622	6.61E-04	0.411417	0.367521
Pdk4	6	2.454545	2.33E-04	0.407407	0.387464
Slc3a2	6	2.837321	0.001168	0.352445	0.233974
Sult5a1	4	3.114833	9.29E-06	0.321045	0.558333
Dio2	3	2.77512	1.11E-05	0.360345	0.5
Ulk1	2	2.909091	0	0.34375	0.607843

### Screening of hub cross talk genes

**Figure 5:**
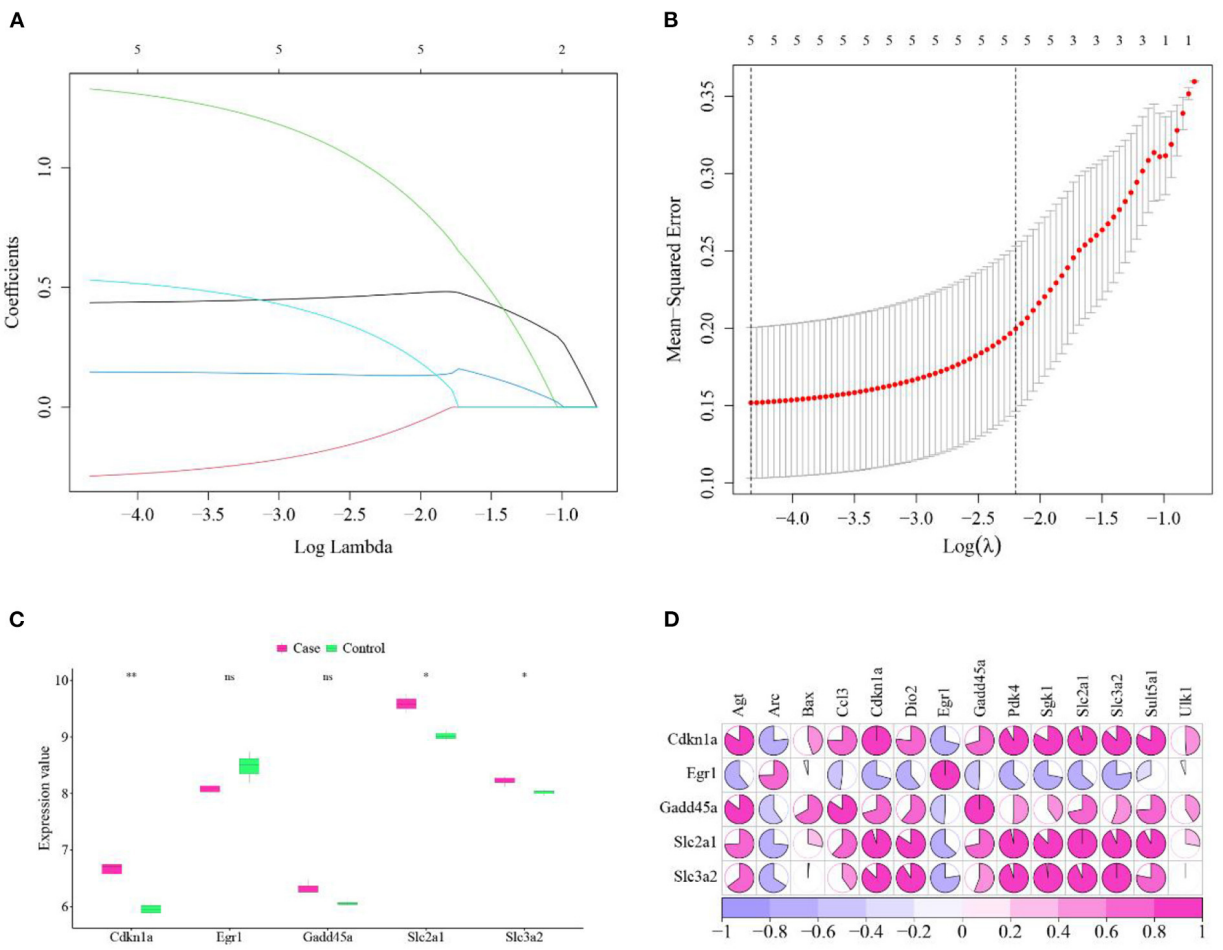
Hub cross talk gene screening and analysis. **(A)** LASSO analysis results, each line in the figure represents a gene. When a gene tends to 0, the larger the value of the abscissa (Log Lambda), the more critical the gene is. **(B)** Results of model cross-validation. There are two dashed lines in the figure, one is the λ value lambda.min when the mean square error is the smallest, and the other is the λ value lambda.1se when the distance from the mean square error is the smallest one standard error, you can choose one of these two values, the dotted line The corresponding number is the result of the number of screening genes, here we choose lambda.1se as the screening condition for key genes. **(C)** Hub cross talk gene expression and *t*-test. The smaller the *P* value value of the test result, the more significant the sample difference results, and the more “*” on the graph. The corresponding relationship between the *P* value and the “*” sign is ns: *P* > 0.05, *: *P* ≤ 0.05, **P ≤ 0.01, ***P ≤ 0.001, ****P ≤ 0.0001. **(D)** Correlation between hub cross talk gene and non-hub cross talk gene.

We used LASSO to perform feature screening on the 14 Cross talk genes (Figures 5A,B), and obtained 5 hub cross talk genes (Cdkn1a, Egr1, Gadd45a, Slc2a1, and Slc3a2). Next, we extracted the expression values of these five hub cross talk genes in cases and controls, and performed *t*-test. The results showed that the expression levels of Cdkn1a, Slc2a1 and Slc3a2 were significantly different between case and control (Figure 5C).

Then we used the Pearson correlation coefficient to analyze the correlation between the hub cross talk genes and the non-hub cross talk genes in gene expression. The gene pairs Slc3a2 and Sgk1, Slc2a1 and Cdkn1a were found highly positive correlated. Slc3a2 and Egr1, Cdkn1a and Arc were negatively correlated (Figure 5D).

### SsGSEA analysis

**Figure 6:**
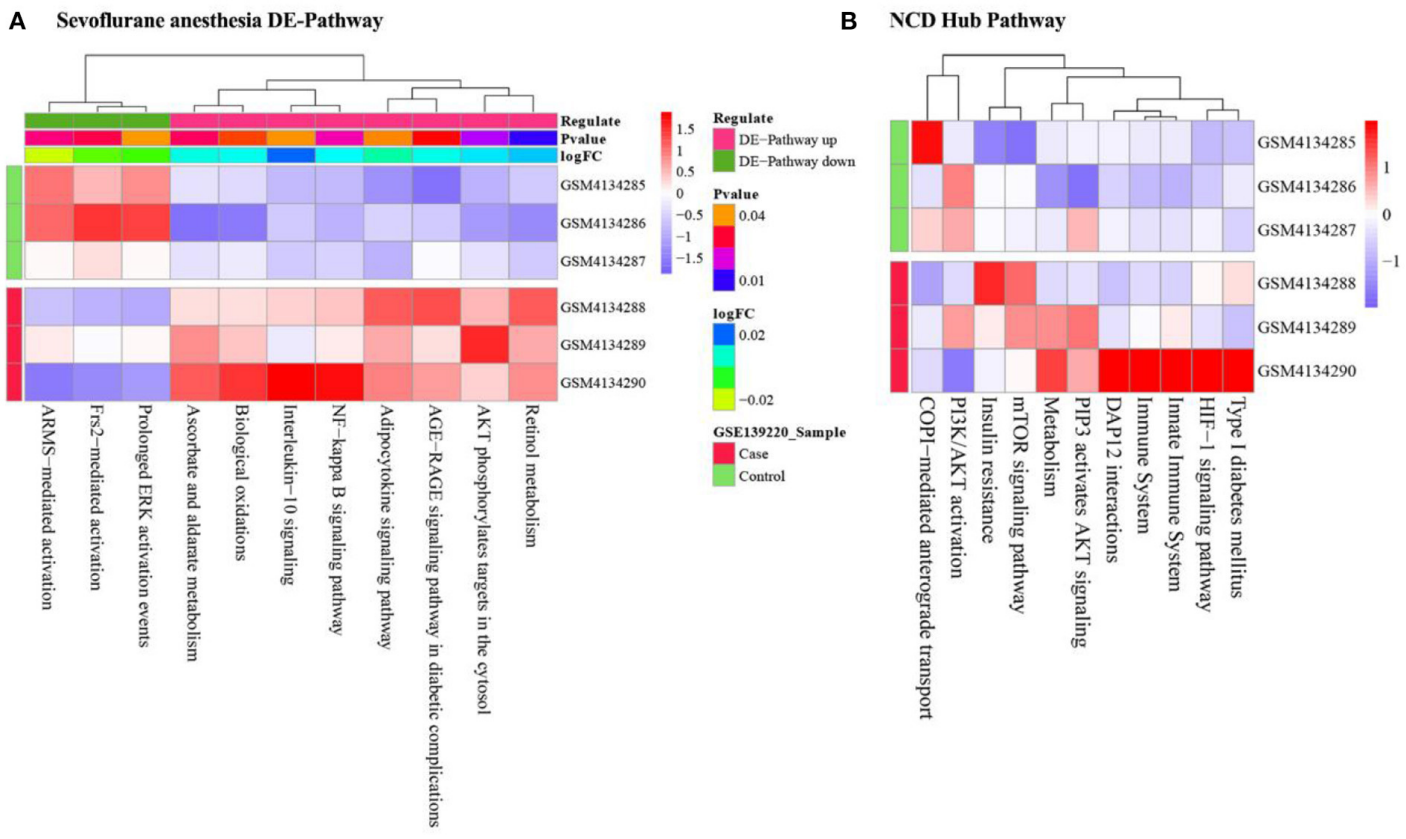
Enrichment abundance of **(A)** Sevoflurane anesthesia DE-pathway and **(B)** NCD hub Pathway.

We obtained all genes under Sevoflurane anesthesia and NCD-related Pathways from KEGG and Reactome, and used ssGSEA to calculate the abundance scores of Sevoflurane anesthesia and NCD-related Pathways in GSE139220, respectively. For the pathways related to Sevoflurane anesthesia, we used the limma package to perform differential expression analysis on the pathway set after ssGSEA analysis, and thus obtained 11 DE-pathways (pathways with *P* value < 0.05 were regarded as DE-PATHWAYS) (Table 3, Figure 6A).

**Table 3:** Sevoflurane anesthesia DE-pathway.

**Pathway**	**LogFC**	***P* value**
Retinol metabolism	0.015774	0.002209
AKT phosphorylates targets in the cytosol	0.013836	0.011622
NF-kappa B signaling pathway	0.012108	0.021568
Ascorbate and aldarate metabolism	0.010656	0.028185
AGE-RAGE signaling pathway in diabetic complications	0.010799	0.035033
Biological oxidations	0.011121	0.03941
Adipocytokine signaling pathway	0.006232	0.044233
Interleukin-10 signaling	0.022627	0.045018
ARMS-mediated activation	−0.0185	0.025965
Frs2-mediated activation	−0.01105	0.029621
Prolonged ERK activation events	−0.0087	0.045874

For NCD-related Pathways, we used the Pearson correlation coefficient to analyze the correlation between Sevoflurane anesthesia DE-pathway and NCD Pathway. The 7 Sevoflurane anesthesia DE-pathways and 11 NCD pathways were found highly correlated (Table 4), and these 11 NCD pathways were marked as NCD hub pathways.

**Table 4:** Correlation of 7 Sevoflurane anesthesia DE-pathways and 11 NCD hub hub pathways.

**Sevoflurane anesthesia_pathway**	**NCD_pathway**	**Correlation**
NF-kappa B signaling pathway	HIF-1 signaling pathway	0.935044
Ascorbate and aldarate metabolism	Metabolism	0.946657
Biological oxidations	Metabolism	0.940878
Interleukin-10 signaling	HIF-1 signaling pathway	0.966138
Interleukin-10 signaling	Type I diabetes mellitus	0.963346
ARMS-mediated activation	HIF-1 signaling pathway	−0.92007
NF-kappa B signaling pathway	Immune system	0.883918
NF-kappa B signaling pathway	Innate immune system	0.862379
NF-kappa B signaling pathway	Type I diabetes mellitus	0.876083
Ascorbate and aldarate metabolism	PIP3 activates AKT signaling	0.850632
AGE-RAGE signaling pathway in diabetic complications	COPI-mediated anterograde transport	−0.89447
AGE-RAGE signaling pathway in diabetic complications	Insulin resistance	0.877234
AGE-RAGE signaling pathway in diabetic complications	mTOR signaling pathway	0.875509
Biological oxidations	Immune system	0.847582
Biological oxidations	Innate immune system	0.869361
Adipocytokine signaling pathway	COPI-mediated anterograde transport	−0.83779
Interleukin-10 signaling	DAP12 interactions	0.826367
Interleukin-10 signaling	Immune system	0.9008
Interleukin-10 signaling	Innate immune system	0.858116
Interleukin-10 signaling	PI3K/AKT activation	−0.84829
ARMS-mediated activation	Type I diabetes mellitus	−0.82896

mTOR signaling pathway, Metabolism and PIP3 activates AKT signaling were highly expressed in NCD disease group (Figure 6B).

### Correlation analysis between hub cross talk genes and pathways

**Figure 7:**
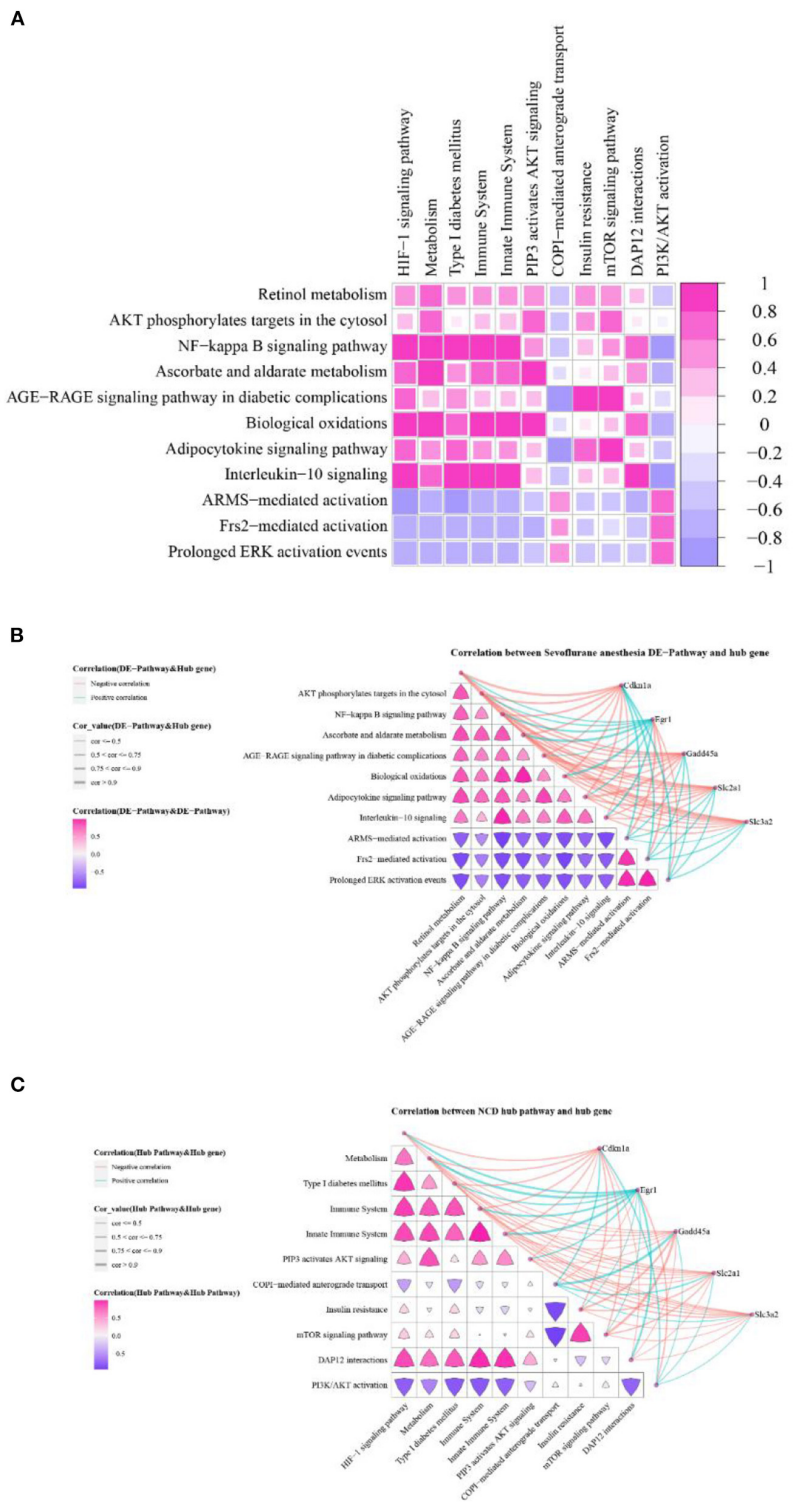
Hub cross talk gene and pathway correlation. **(A)** Correlation of Sevoflurane anesthesia DE-pathway and NCD hub pathway. **(B)** Correlation between Sevoflurane anesthesia DE-pathway, and hub cross talk gene and Sevoflurane anesthesia DE-pathway. **(C)** The correlation between the NCD hub pathway and the hub cross talk gene and the NCD hub pathway.

We calculated the correlation between the Sevoflurane anesthesia DE-pathway and the NCD hub Pathway using the Pearson correlation coefficient. The results showed that Interleukin-10 signaling and HIF-1 signaling pathway, NF-kappa B signaling pathway and HIF-1 signaling pathway, Biological oxidations and Metabolism were highly correlated (Figure 7A).

We analyzed the relationship among Sevoflurane anesthesia DE-pathways. The results showed that these pathway pairs were highly correlated: Frs2-mediated activation and Prolonged ERK activation events, ascorbate and aldarate metabolism and Biological oxidations, NF-kappa B signaling pathway and Interleukin-10 signaling, Prolonged ERK activation events ARMS and mediated activation (Figure 7B). At the same time, we analyzed the relationship between Sevoflurane anesthesia DE-pathway and hub cross talk genes. Results showed that Egr1 was highly correlated with Biological oxidations, Ascorbate and aldarate metabolism, and NF-kappa B signaling pathway (Figure 7B).

Furthermore, we analyzed the relationship among NCD hub pathways. The results showed that Immune System and DAP12 interactions, Immune System and HIF-1 signaling pathway, Insulin resistance m and TOR signaling pathway were highly correlated (Figure 7C). Finally, we analyzed the relationship between these NCD hub pathways and hub cross talk genes, and obtained high correlation between Egr1 and Innate Immune System and Metabolism (Figure 7C).

### The relationships between hub cross talk genes, target genes and pathways

**Figure 8:**
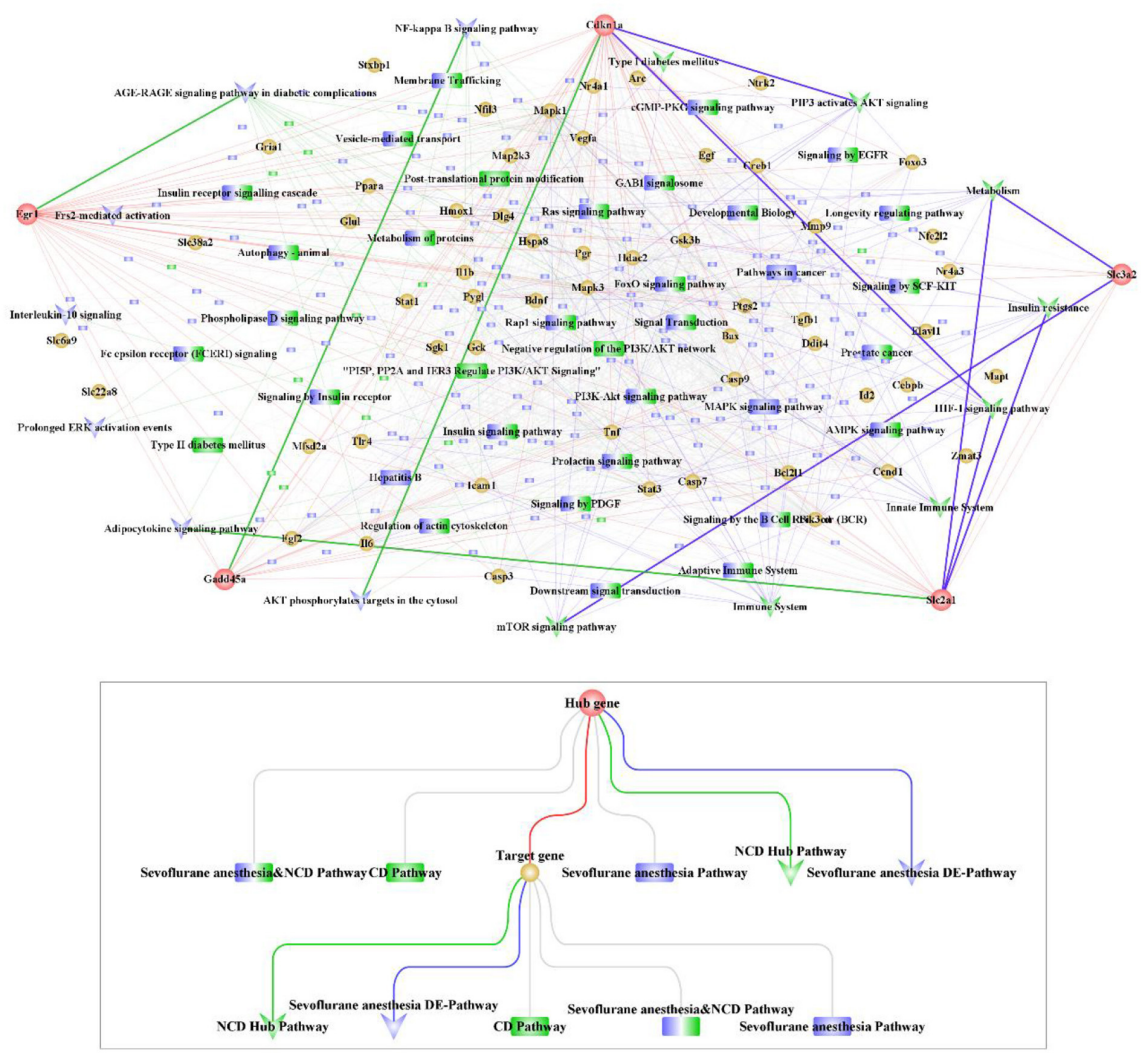
Hub cross talk gene-Target gene-pathway network analysis. The network contains 281 nodes and 1,328 edges, including 5 hub cross talk genes, 55 Target genes, 160 Sevoflurane anesthesia Pathway, 18 NCD Pathway, 28 Sevoflurane anesthesia & NCD Pathway, 8 NCD hub Pathway and 7 Sevoflurane anesthesia DE-pathway.

We obtained the target genes that interacted with the five hub cross talk genes from the PPI of the cross talk gene. Then we extracted the related pathways of hub cross talk gene and target gene in Sevoflurane anesthesia. In addition, we extracted the related pathways of hub cross talk gene and target gene in NCD. Based on the above relationship pairs, we constructed the hub cross talk gene-target gene-pathway network (Figure 8).

The results showed that Egr1 interacted with AGE-RAGE signaling pathway in diabetic complications, Apelin signaling pathway, Cdkn1a and Gadd45a. Furthermore, Cdkn1a was involved in FoxO signaling pathway, PI3K-Akt signaling pathway, ErbB signaling pathway and Oxytocin signaling pathway. In addition, Cdkn1a interacted with Gadd45a. Moreover, Gadd45a was involved in the regulation of NF-kappa B signaling pathway and FoxO signaling pathway.

### Candidate drug prediction

**Figure 9:**
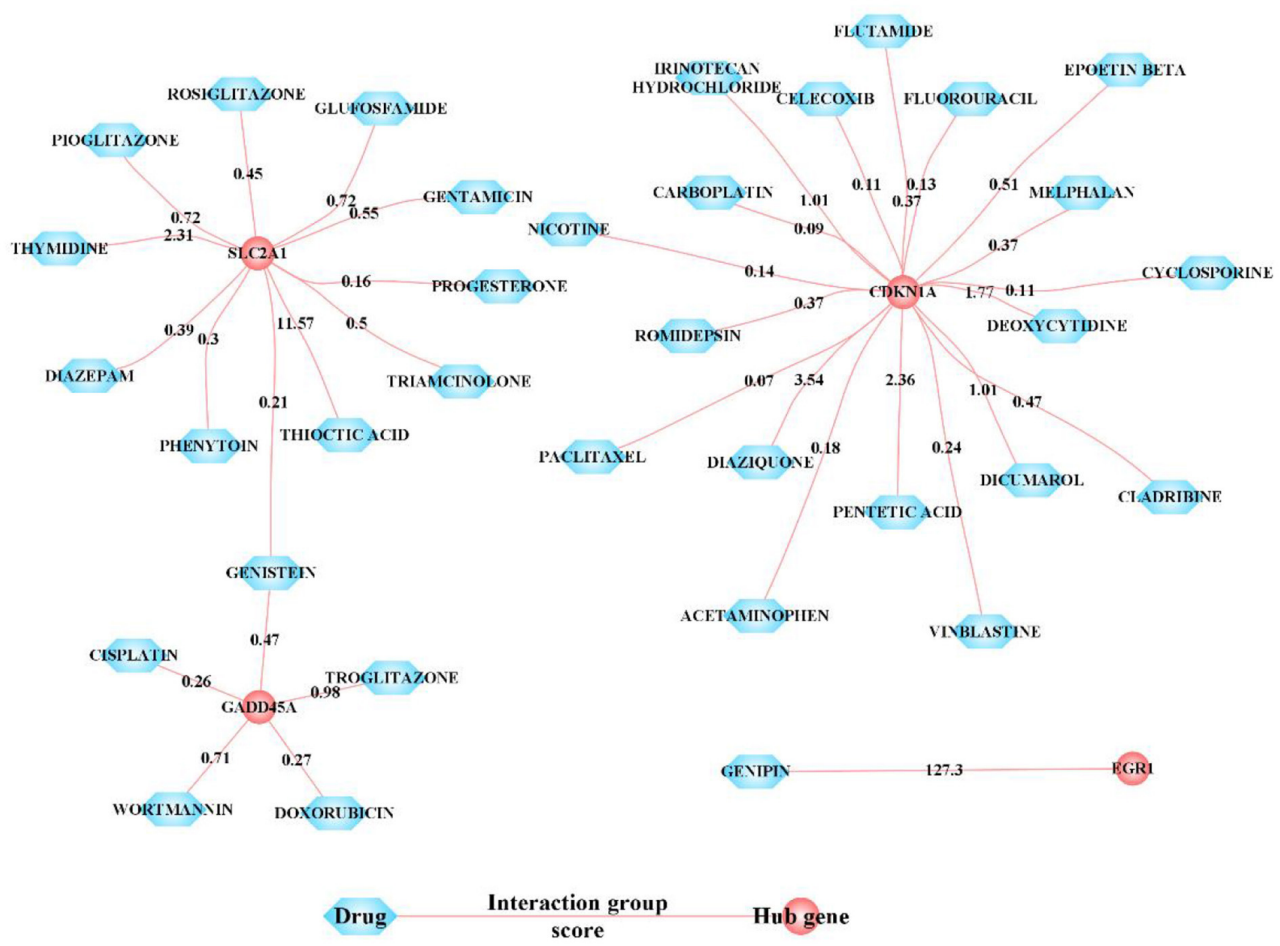
Sensitivity relationship between hub cross talk gene and Drug. In the network, nodes with interaction group score ≥4 are displayed, and other nodes are hidden. The interaction score is a static score and based on the evidence of an interaction.

We extracted the sensitive drugs of the hub cross talk gene from the DGIdb database, and obtained 4 hub cross talk genes that are sensitive to drugs. We constructed hub cross talk gene-Drug network for hub cross talk gene and drug (Figure 9). As a result, Egr1 and the drug GENIPIN were obtained with high sensitivity. In addition, Gadd45a and Cdkn1a are sensitive to various drugs.

## Discussion

We applied a series of bioinformatic analysis including feature selection among cross-talk genes and composite network analysis to identify candidate genes and functional pathways that might mediate Sevoflurane anesthesia associated cognitive disturbance and neurological injury. 14 cross talk genes were identified, among which 5 genes, Cdkn1a, Egr1, Gadd45a, Slc2a1 and Slc3a2, emerged as ‘hub' genes, suggesting highly relevant mechanistic roles. Egr 1 also showed a central role in the PPI network.

The Egr1 ‘Early Growth Response 1' gene is a transcriptional regulator, an ‘early gene' induced rapidly in response to stimulus and Egr1 is widely documented in the nervous system as responsive to oxidative stress (22). The Egr1 gene is well established in playing a role in hippocampal functions including memory acquisition and potentiation (23). In the hippocampus, the GABA_A_ receptor subunits are documented as transcriptional targets of Egr1, which regulates their composition (24), which is consistent with our findings. Animal experiments have indicated a key role of Egr1 in Propofol anesthesia mediated cognitive dysfunction (25, 26). Sevoflurane has been shown to depress Egr1 expression during the anesthetic and post anesthetic period (27). Our results showed Egr1 was implicated in AGE RAGE signaling in context of Sevoflurane related neurotoxicity. The role of RAGE (receptor for advanced glycation end-products) in regulating the blood brain barrier is established along with a protective role of anti-RAGE antibody in Isoflurane related cognitive dysfunction (28). Sevoflurane can disrupt the barrier by damage to brain vascular endothelial cells (29) but the specific role of AGE-RAGE signaling is yet to be comprehensively described. Egr1 was also implicated in Apelin signaling, Apelin being an orphan G-protein-coupled receptor APJ ligand with anti neuronal apoptotic and neuroprotective roles (30).

Cdkn1a is a cell cycle arrest and senescence marker gene targeted by the p53 gene (31). In Alzheimer's disease Cdkn1a expression marks senescence like phenotype and inflammation (32). Sevoflurane anesthesia reportedly can increase p53 signaling along with rise in the apoptosis marker Caspase-3 (33). The solute carrier transporters (Slc), Slc2a1 and Slc3a2 comprised hub cross talk genes. Slc genes are regulators of the blood brain barrier (34). The potential mechanistic role of Sevoflurane in inducing Slc gene mediated disruption of the blood brain barrier remains to be well investigated. The hub gene Gadd45a was found involved in NF-kappa B signaling and FoxO signaling pathways. Gadd45a (growth arrest and DNA damage-inducible protein 45 alpha) has been found upregulated in response to Sevoflurane along with the corresponding long noncoding RNA (35) and also activates the p53 pathway. Gadd45a is implicated in hippocampal memory and potentiation (36). Composite network analysis implicated Gadd45a, a DNA-damage-inducible gene, in modulating NF-kappa B signaling in Sevoflurane related cognitive damage. In cancer, Gadd45a was found induce Gadd45a-dependant apoptosis in response to NF-kappa B signaling (37) and serves as important links between NF-kappa B and MAPK signaling (38). Sevoflurane was found to impair the AMPK/FoxO3a signaling pathway activation leading to apoptosis of mouse hippocampal neurons (39) and in mouse cerebral cortex (40). FoxO signaling is implicated in increased M1 type polarization of macrophages in Sevoflurane related postoperative cognitive dysfunction (41). Sevoflurane and NCD related pathways were found highly correlated and largely supported by existing literature. NF-kappa B was highly correlated with HIF-1 α in context of Sevoflurane related NCD. HIF 1 α (Hypoxia-inducible factor-1α) can be activated by inhalational anesthetics and is implicated in the disruption of the blood brain barrier in postoperative cognitive dysfunction induced by Isoflurane (42). We also performed drug sensitivity analysis for the hub genes and found a number of candidate drugs. Genipin, a biological compound derived from *Gardenia jasminoides* was found to target Erg1, a key candidate gene. Earlier research has shown Genipin could modulate the levels of 5-HT genes and BDNF in the hippocampus (43). The present data suggests the roles of Apexin and Genipin among others to counter Sevoflurane related cognitive dysfunction. Several drugs including dexmedetomidine, ketamine, other anti-inflammatory and anti-oxidant molecules (44, 45) have been documented for treatment of post-operative cognitive dysfunction. Our analysis identified several anti-diabetic drugs as candidates, which have shown potential in Alzheimer's disease (46) but are not documented for post-operative cognitive protection. In addition progesterone, which has been documented for post brain surgery cognitive dysfunction (47) was also identified as a candidate.

Overall the candidate genes and functional pathways implicated in Sevoflurane related postoperative cognitive dysfunction was supported by existing experimental data. Although earlier studies have utilized gene expression analysis for analysis of postoperative cognitive dysfunction (48, 49) no studies have focused on Sevoflurane to our knowledge. Furthermore, as many peri-operative factors can contribute to postoperative cognitive dysfunction, it is challenging in to isolate molecular mechanisms attributable to an anesthetic agent alone. By the *in-silico* approach adopted in this study, overlapping mechanisms were identified as candidates attributed to the anesthetic agent. Future studies should focus on verifying the roles of specific candidate gene-signaling axis pathways that might mediate Sevoflurane related postoperative cognitive dysfunction. The mechanism of postoperative cognitive dysfunction is complex. Neuroimaging studies have identified the role of other areas such as the thalamus (50), which were not addressed in the present study. While the present data are limited by lack of experimental verification, they offer a sound preliminary basis for directing experimental and clinical research concerning Sevoflurane related postoperative cognitive dysfunction.

## Conclusion

A suite of bioinformatics analysis revealed several key candidate hippocampal genes including Cdkn1a, Egr1, Gadd45a, Slc2a1 and Slc3a2 and multiple enriched functional signaling pathways that could underlie Sevoflurane induced neurodegenerative processes.

## Data availability statement

The data presented in the study are deposited in the GEO dataset repository, accession number GSE139220 dataset (URL: https://www.ncbi.nlm.nih.gov/geo/query/acc.cgi?acc=GSE139220). The original contributions presented in the study are included in the article, further inquiries can be directed to the corresponding author.

## Ethics statement

Ethical review and approval was not required for the study on animals in accordance with the local legislation and institutional requirements. Ethical review and approval was not required for the study on human participants in accordance with the local legislation and institutional requirements. Written informed consent from the patients/participants' or patients/participants legal guardian/next of kin was not required to participate in this study in accordance with the national legislation and the institutional requirements.

## Author contributions

WL and QY conceptualized the research idea, designed the workflow, carried out the computational biology analysis, interpreted the results, and wrote the draft of the manuscript. HS reviewed and edited the manuscript, as well as administrated and supervised the whole research project. All authors read and approved the final manuscript.

## Funding

We appreciate the research funding provided by the Science and Technology Innovation Development Project of Tai'an (Grant No. 2021NS160), the Medical and Health Science and Technology Development Plan of Shandong Province (Grant No. 202102010647), the Traditional Chinese Medicine Science and Technology Project of Shandong Province (Grant No. 2021M190), and the Science and Technology Innovation Development Project of Tai'an (Grant Nos. 2020NS188 and 2020NS217).

## Conflict of interest

The authors declare that the research was conducted in the absence of any commercial or financial relationships that could be construed as a potential conflict of interest.

## Publisher's note

All claims expressed in this article are solely those of the authors and do not necessarily represent those of their affiliated organizations, or those of the publisher, the editors and the reviewers. Any product that may be evaluated in this article, or claim that may be made by its manufacturer, is not guaranteed or endorsed by the publisher.
